# Genetic Characterization of Cancer of Unknown Primary Using Liquid Biopsy Approaches

**DOI:** 10.3389/fcell.2021.666156

**Published:** 2021-06-10

**Authors:** Noemi Laprovitera, Irene Salamon, Francesco Gelsomino, Elisa Porcellini, Mattia Riefolo, Marianna Garonzi, Paola Tononi, Sabrina Valente, Silvia Sabbioni, Francesca Fontana, Nicolò Manaresi, Antonia D’Errico, Maria A. Pantaleo, Andrea Ardizzoni, Manuela Ferracin

**Affiliations:** ^1^Department of Experimental, Diagnostic and Specialty Medicine (DIMES), University of Bologna, Bologna, Italy; ^2^Department of Life Sciences and Biotechnologies, University of Ferrara, Ferrara, Italy; ^3^Center for Applied Biomedical Research (CRBA), University of Bologna, Italy; ^4^Divisione di Oncologia Medica, IRCCS Azienda Ospedaliero-Universitaria di Bologna, Bologna, Italy; ^5^Pathology Unit, Sant’Orsola Hospital, IRCCS Azienda Ospedaliero-Universitaria di Bologna, Bologna, Italy; ^6^Menarini Silicon Biosystems S.p.A, Bologna, Italy

**Keywords:** CTC, cell-free tumor DNA, cancer of unknown primary, liquid biopsy, precision oncology

## Abstract

Cancers of unknown primary (CUPs) comprise a heterogeneous group of rare metastatic tumors whose primary site cannot be identified after extensive clinical–pathological investigations. CUP patients are generally treated with empirical chemotherapy and have dismal prognosis. As recently reported, CUP genome presents potentially druggable alterations for which targeted therapies could be proposed. The paucity of tumor tissue, as well as the difficult DNA testing and the lack of dedicated panels for target gene sequencing are further relevant limitations. Here, we propose that circulating tumor cells (CTCs) and circulating tumor DNA (ctDNA) could be used to identify actionable mutations in CUP patients. Blood was longitudinally collected from two CUP patients. CTCs were isolated with CELLSEARCH^®^ and DEPArray^TM^ NxT and Parsortix systems, immunophenotypically characterized and used for single-cell genomic characterization with *Ampli*1^TM^ kits. Circulating cell-free DNA (ccfDNA), purified from plasma at different time points, was tested for tumor mutations with a CUP-dedicated, 92-gene custom panel using SureSelect Target Enrichment technology. In parallel, FFPE tumor tissue was analyzed with three different assays: FoundationOne CDx assay, DEPArray LibPrep and OncoSeek Panel, and the SureSelect custom panel. These approaches identified the same mutations, when the gene was covered by the panel, with the exception of an insertion in *APC* gene. which was detected by OncoSeek and SureSelect panels but not FoundationOne. *FGFR2* and *CCNE1* gene amplifications were detected in single CTCs, tumor tissue, and ccfDNAs in one patient. A somatic variant in *ARID1A* gene (p.R1276^∗^) was detected in the tumor tissue and ccfDNAs. The alterations were validated by Droplet Digital PCR in all ccfDNA samples collected during tumor evolution. CTCs from a second patient presented a pattern of recurrent amplifications in *ASPM* and *SEPT9* genes and loss of *FANCC*. The 92-gene custom panel identified 16 non-synonymous somatic alterations in ccfDNA, including a deletion (I1485Rfs^∗^19) and a somatic mutation (p. A1487V) in *ARID1A* gene and a point mutation in *FGFR2* gene (p.G384R). Our results support the feasibility of non-invasive liquid biopsy testing in CUP cases, either using ctDNA or CTCs, to identify CUP genetic alterations with broad NGS panels covering the most frequently mutated genes.

## Introduction

Malignant solid tumors that are not fully eradicated at an early stage are doomed to spread to nearby lymph nodes and organs and eventually give origin to distant metastases. A diagnosis of metastatic cancer considerably reduces the chances to eliminate the tumor and cure the patient. The metastatic process is made possible by the progressive acquirement of genetic alterations in tumor cells, which eventually become anchorage independent and self-sufficient for cell growth. Disseminated tumor cells, including circulating tumor cells (CTCs) and CTC clusters, are highly metastatic (Aceto et al., 2014), and their number in the blood correlates with patient prognosis in several cancer types (Riethdorf et al., 2007; Joosse et al., 2015; Sparano et al., 2018).

The epitome of advanced cancers could be considered a particularly aggressive metastatic disease known as cancer of unknown primary (CUP, also occult primary cancer). CUP is a rare syndrome of metastatic cancers whose primary site cannot be identified after detailed physical examinations, blood analyses, imaging, and immunohistochemical (IHC) testing (Fizazi et al., 2015). CUPs make up 3–5% of newly diagnosed cancers worldwide and represent an important clinical problem (Varadhachary and Raber, 2014; Laprovitera et al., 2021), specifically when considering that current therapeutic protocols are primary site oriented. As a final result, CUP patients receive empirical cytotoxic chemotherapy regimens and have poor prognosis [average overall survival (OS) 4–9 months, about 20% survive more than 1 year]. It has been reported that CUPs present actionable genetic alterations (Ross et al., 2015; Varghese et al., 2017), and these alterations can be identified in circulating tumor DNA (ctDNA) (Kato et al., 2017). Therefore, an improvement in CUP treatment and outcome could derive from clinical trials where patients are treated in accordance to their specific actionable mutations (Conway et al., 2019), as in NCT02628379 based on FoundationOne CDx target sequencing (Roche Foundation Medicine), or basket trials extended to CUP patients. Moreover, recent studies evidenced how CUP patients could benefit from treatment with immune checkpoint inhibitors (Haratani et al., 2019), and clinical trials have been opened and are currently recruiting (NCT04131621) or positively concluded (UMIN000030649). In this clinical trial, 56 CUP patients, of whom 45 previously treated, were treated with anti PD-1 nivolumab; the objective response rate (ORR) in the overall population was 21.4%, with a median progression-free survival (PFS) and OS of 5.1 (95% CI, 2.7–5.6) and 15.9 months (95% CI, 8.4–not reached), respectively (Tanizaki et al., 2020).

CUP genetic testing is currently limited by the scarcity of tumor biopsy material and reduced biopsy DNA quality; therefore, a liquid biopsy approach could be of the outmost interest. Several works demonstrated the utility of ctDNA and CTC testing to monitor cancer patient prognosis and guide treatment decisions (Ignatiadis and Dawson, 2014). In addition, ctDNA variant allele frequency was found to be associated with a worse prognosis in patients with metastatic disease (Pairawan et al., 2020); moreover, CTCs became an important clinical prognostic biomarker (Amintas et al., 2020) with potential application in *in vitro* (Zhao et al., 2019) and *in vivo* disease modeling (Drapkin et al., 2018) and drug testing (Yu et al., 2014). Size-based or antigen-based technologies for CTC isolation and/or enumeration have been developed in the past 10 years, each one presenting advantages and limitations (Yu et al., 2011).

CUP patients are usually diagnosed with an advanced metastatic disease; therefore, they are likely to have a high number of CTCs and CTC clusters in the circulation. Given the CUP undifferentiated status and variable presentation, it is yet to demonstrate whether CUP CTCs could be isolated using tumor antigen selection (Komine et al., 2014). In this study, we explored liquid biopsy, specifically ctDNA- and CTC-based applications, as approaches to detect CUP druggable mutations. We compared two methods to isolate CTCs, one antigen-based, size-agnostic (CELLSEARCH, Menarini Silicon Biosystems) and another antigen-agnostic, size-based (Parsortix, ANGLE plc). CTCs and ctDNA were detectable in the blood of CUP patients and analyzed for genomic alterations, which were further compared with genomic alterations identified in tumor biopsy.

## Materials and Methods

### Sample Collection

Two patients (Pt#71 and Pt#95) with a diagnosis of cancer of unknown origin (CUP) were recruited at Bologna University Hospital, Italy. The study was conducted in accordance with the Declaration of Helsinki, and the protocol was approved by the Ethics Committee Center Emilia-Romagna Region—Italy (protocol 130/2016/U/Tess). Patients provided written informed consent.

Metastatic tissue from lymph node (Pt#71) and ampulla of Vater (Pt#95) was formalin-fixed and paraffin-embedded (FFPE) and used for tumor DNA collection. For Pt#71, blood sampling was performed at three different time points: (A) at diagnosis (August 2018), (B) during FOLFOX-4 treatment (stable disease, November 2018), and (C) at disease progression (May 2019). For Pt#95, blood sampling was performed at diagnosis.

Plasma separation was performed *via* centrifugation at 1,900 × *g* for 10 min at 4°C. A variable number (*N* = 2–5) of plasma aliquots (1 ml) for each patient was collected and stored at –80°C prior to isolation of circulating cell-free DNA (ccfDNA). PBMCs were isolated from peripheral blood of Pt#95 using Ficoll-Paque Plus (17-1440-02, GE Healthcare, Chicago, IL, United States). Briefly, after plasma depletion, an equal volume of PBS was added to the remaining blood in EDTA tubes. Following, 4 ml of diluted blood was stratified on 3 ml of Ficoll-Paque Plus and centrifuged at 400 × *g* for 30 min at room temperature in a swinging-bucket rotor. The PBMC ring was carefully removed by pipetting and the pellet was washed with PBS and centrifuged at 100 × *g* for 5 min. Finally, the pellet was stored at –80°C prior to proceeding with DNA extraction.

### CTC Isolation With CELLSEARCH and DEPArray NxT

Pt#71 and Pt#95 blood samples were collected at diagnosis. CELLSEARCH^®^ system (Menarini Silicon Biosystems, Castel Maggiore, Italy) was used to enrich and enumerate CTCs, followed by single-cell isolation with the DEPArray^®^ NxT system (Menarini Silicon Biosystems). Briefly, 7.5 ml of blood was loaded in CELLSEARCH Autoprep^®^ for EpCAM-based immunomagnetic capture of CTCs, followed by immunofluorescent staining (CK-FITC, PD-L1-PE, CD45-APC, and DAPI using the CELLSEARCH CXC kit in one patient, and CK-PE, CD45-APC, and DAPI with the CELLSEARCH CTC kit in the other patient). After CTC enumeration with the CellTrack Analyzer II, the sample was loaded into a DEParray Cartridge and single CTCs and leukocytes were isolated with the DEPArray NxT system (Menarini Silicon Biosystems) from each sample. A variable number of CTCs and leukocytes, used as control, were isolated as single cells and then subjected to whole-genome amplification employing the *Ampli*1 WGA Kit (Menarini Silicon Biosystems).

### CTC Isolation With the Parsortix System

For Pt#95, tumor cells were enriched from peripheral blood also using the Parsortix system (ANGLE plc, Guildford, United Kingdom). Blood cells were forced to pass through a 6.5-μm separator cassette that can capture CTCs based on their size and deformability properties. Then, to confirm the CTC nature of retained cells, we stained CTCs using a combination of anti-human EpCAM-FITC, anti-human CD45-APC (BD 561864/555485), and DAPI for cell nuclei staining. Images were acquired using a Leica DMI6000 B inverted fluorescence microscope (Leica Microsystems, Wetzlar, Germany).

### DNA Extraction

DNA was extracted from the tumor area after selection by an expert pathologist, with the guidance of the hematoxylin–eosin-stained section. The DNA from different areas of FFPE unstained slides was extracted using the QIAamp DNA FFPE Tissue Kit (Cat No: 56404, Qiagen, Hilden, Germany) following the manufacturer’s guidelines (as described in Cinausero et al., 2019). Normal DNA was extracted from a non-tumor area in formalin-fixed paraffin-embedded (FFPE) tissue for Pt#71 and from PBMC for Pt#95. DNA from PBMCs was extracted using the QIAamp DNA Micro kit (Cat No: 56304, Qiagen, Hilden, Germany). Ten slices of Pt#71 FFPE tumor tissue were sent to Roche Foundation Medicine (Cambridge, MA, United States) for comprehensive genomic profile analysis with FoundationOne CDx (F1CDx) assay.

ccfDNA was purified from 1 ml of plasma by Maxwell RSC instrument (Cat No: AS4500, Promega, Madison, WI, United States) using the Maxwell RSC ccfDNA Plasma Kit (Cat No: AS1480, Promega). Genomic and ccfDNA were quantified using the Qubit 4.0 Fluorometer (Cat No: Q33238, Thermo Fisher Scientific, Waltham, MA, United States) and the Qubit dsDNA High Sensitivity Assay Kit (Cat No: Q32854, Thermo Fisher Scientific).

We assessed yields and quality of FFPE-derived gDNAs using the NGS FFPE QC Kit (Cat No: G9700B, Agilent Technologies, Santa Clara, CA, United States) according to the producer’s protocol (G9700-90000, v. E1, May 2018, Agilent Technologies).

### Analysis of Tumor Genetic Alterations

A 1.2-Mb custom SureSelect capture bait library (Agilent Technologies, Santa Clara, CA, United States) was designed to target 92 genes (listed in Supplementary Table 1), selected to be the most frequently mutated genes in CUPs, as reported in MSK Impact study CUP cohort (Zehir et al., 2017), including druggable or potentially actionable alterations. The panel was designed using Agilent SureDesign web application (v. 7.0, Agilent Technologies)^1^ to cover coding exons and UTRs, with 25 flanking bases from 3′ and 5′ ends. This panel comprehends up to 51,466 probes included in five probe groups, with 2 × tiling density, balanced boosting, and 86% of probes with the most stringent masking. Libraries were prepared using the custom gene panel following *SureSelectXT HS/SureSelectXT Low Input Target Enrichment with Pre-Capture Pooling* protocol (G9702-90005, v. A0, June 2019, Agilent Technologies). An input of 50 ng of gDNA (normal and tumor) underwent enzymatic fragmentation according to *SureSelect Enzymatic Fragmentation* protocol (G9702-90050, Revision B0, January 2020, Agilent Technologies). Libraries from ccfDNAs of Pt #71 (A, C) and Pt#95 were prepared starting from 25 ng of DNA bypassing the fragmentation step, as described in a published application note (Barber et al., 2017). Libraries were sequenced on NextSeq 500 (Illumina) platform using High Output 2 × 75-bp flow cells. Variant calling and paired analyses (tumor vs. normal) were performed using SureCall software (v. 4.2), applying a filter for all samples at 5%, and at 1% for ccfDNAs. Somatic alterations were filtered to only keep exonic non-synonymous single-nucleotide variants (SNVs), insertions, deletions, multiple nucleotide variants, and long deletions not detected in the normal sample.

Alterations with population allele frequency (Genome Aggregation Database, GnomAD; Karczewski et al., 2020) higher than 0.5% in Non-Finnish European (NFE) population were also filtered out. All variants reported had a coverage higher than 100. Target sequencing data (92-gene custom panel) were used to assess copy number variants using panelcn. MOPS pipeline (Povysil et al., 2017). Variants triage and clinical classification was obtained with Alissa Interpret software (Agilent Technologies). Variants were also annotated using ANNOVAR (Wang et al., 2010). Bioinformatic pathogenicity prediction, reported in Tables 1, 2, of the identified variants was performed consulting the prediction score/outcome of SIFT (Sort Intolerated From Tolerated; Vaser et al., 2016), Polyphen2 HVAR (Polymorphism Phenotyping v2; Adzhubei et al., 2010), LRT (Likelihood Ratio Test; Chun and Fay, 2009), Mutation Taster (Schwarz et al., 2014), Mutation Assessor (Reva et al., 2011), FATHMM (Functional Analysis Through Hidden Markov Model; Shihab et al., 2013), CADD (Combined Annotation Dependent Depletion; Kircher et al., 2014), and VEST (Variant Effect Scoring Tool; Douville et al., 2016). Genes with evidence for actionability and the FDA-approved drugs reported in Tables 1, 2 were obtained consulting OncoKB (Chakravarty et al., 2017).

**TABLE 1:** Mutation report of tumor, ccfDNA, and CTCs from Pt#71.

				**SureSelect 92-gene panel (Agilent Technologies)**	**SureSelect 92-gene panel (Agilent Technologies)**	**SureSelect 92-gene panel (Agilent Technologies)**	**Foundati-One CDx (Roche)**	**Ampli1(^TM^ OncoSeek and LowPass Kit (Menarini Silicon Biosystem)**	**DEPArray(^TM^ OncoSeek and LowPass Kit (Menarini Silicon Biosystem)**	**Pathogenicity Prediction**								**Actional genes/approved drugs [OncoKB (Chakravarty et al., 2017)]**
**Gene**	**Position**	**Coding change**	**Amino acidic change**	**Tumor FFPE**	**ccfDNA at diagnosis**	**ccfDNA at PD**	**Tumor FFPE**	**CTCs**	**Tumor FFPE**	**SIFT (Vaser et al., 2016)**	**Polyphen2 (Adzhubei et al., 2010)**	**LRT (Chun and Fay, 2009)**	**Mutation Taster (Schwarz et al., 2014)**	**Mutation Assessor (Reva et al., 2011)**	**FATHMM (Shihab et al., 2013)**	**CADD (Kircher et al., 2014)**	**VEST3 (Douville et al., 2016)**	
APC	chr5:112840254	4607insA	p.T1556fs*3	9.8%	22.6%	29.9%	ND	90.2%-99.2%	14.0%	–	–	–	–	–	–	–	–	–
ARID1A	chr1:26773456	c.C3826T	p.R1276X	8.9%	28.5%	22.8%	7.3%	NI	NI	–	–	–	DC	–	–	HF	–	–
ERBB3	chr12:56085144	c.C384A	p.S128R	9.8%	20.7%	20.4%	8.5%	NI	NI	–	PS	DT	DC	NFL	TL	BG	HF	–
NTRK1	chr1:156881475	C2206T	p.Q736X	8.3%	26.0%	22.3%	7.6%	NI	NI	DT	–	DT	DC	–	–	HF	–	–
KEAP1	chr19:10499630	c.G404A	p.R135H	9.0%	22.6%	19.5%	7.0%	NI	NI	TL	PD	DT	DC	NFL	TL	HF	HF	–
PALB2	chr16:23629735	C2419T	p.P807S	9.0%	19.1%	16.6%	6.0%	NI	NI	TL	BG	NL	PM	NFN	TL	BG	BG	Level 1/Olaparib*
KMT2C	chr7:152265209	c.C1013T	p.S338L	ND	5.5%	4.3%	NI	NI	NI	TL	PD	U	DC	NFL	DG	BG	BG	–
KMT2C	chr7:152247975	c.C2459T	p.T820I	ND	1.1%	1.1%	NI	NI	NI	DT	PS	DT	DC	NFL	DG	HF	HF	–
PTPRD	chr9:8404618	c.T2908G	p.L970V	ND	5.1%	3.5%	NI	NI	NI	–	PD	DT	DC	FCM	TL	BG	HF	–
TP53	chr9:8404618	c.T316C	p.C106R	ND	2.1%	ND	ND	ND	ND	DT	PD	DT	DC	FCM	DG	HF	HF	Levels 1–3/no drug
TP53	chr17:7674251	c.G315C	p.M105I	ND	1.8%	ND	ND	ND	ND	DT	PD	DT	DC	FCM	DG	HF	HF	Levels 1–3/no drug
TP53	chr17:7674252	c.A355G	p.I119V	ND	1.7%	ND	ND	ND	ND	DT	PD	DT	DC	FCM	DG	HF	HF	Levels 1–3/no drug
TP53	chr17:7674212	c.C9G	p.C3W	ND	1.2%	ND	ND	ND	ND	DT	PD	DT	DC	FCM	DG	BG	HF	Levels 1–3/no drug
ALK	chr2:29328463	c.A1301G	p.K434R	ND	ND	7.0%	ND	OTR	OTR	–	BG	U	DC	NFN	TL	HF	BG	Level 1/Lorlatinib, Brigatinib*
EPHA5	chr4:65420551	c.A1417C	p.T473P	ND	ND	8.3%	NI	NI	NI	TL	PS	NL	DC	NFL	TL	BG	HF	–
FAT1	chr4:186707744	c.A2084C	p.N695T	ND	ND	2.6%	NI	NI	NI	TL	BG	NL	PM	NFN	TL	BG	BG	–
MGA	chr15:41736268	c.T4004C	p.L1335P	ND	ND	1.6%	NI	NI	NI	DT	PD	NL	DC	NFN	TL	BG	HF	–
KMT2C	chr7:152248206	c.C2228T	p.P743L	ND	ND	1.6%	NI	NI	NI	TL	BG	NL	PM	NFN	DG	BG	BG	–
KMT2C	chr7:152235897	c.C2689T	p.R897X	ND	ND	1.4%	NI	NI	NI	–	–	–	DC	–	–	HF	–	–
PTPRD	chr9:8389280	c.A3117C	p.Q1039H	ND	ND	6.8%	ND	NI	NI	–	PD	DT	DC	FCM	DG	BG	HF	–
ABL1	chr9:133760790	c.C3113T	p. A1038V	NI	NI	NI	52.8%	OTR	OTR	–	BG	–	–	NFN	–	HF	–	–
GRM3	chr7:86415916	c.C808T	p.R270C	NI	NI	NI	7.0%	NI	NI	–	PS	–	–	FCM	–	HF	–	–
MAP3K1	chr5:56177843	c.C2816G	p.S939C	NI	NI	NI	49.6%	NI	NI	–	BG	–	–	–	–	HF	–	–
MSH3	chr5:80149992	c.A2857T	p.M953L	NI	NI	NI	46.1%	NI	NI	–	PD	–	–	FCM	–	HF	–	–
MSH6	chr2:48028273	c.G3151A	p.V1051I	NI	NI	NI	50.0%	OTR	OTR	–	BG	–	–	NFL	–	BG	–	–
PTPRO	chr12:15475691	c.G31T	p.A11S	NI	NI	NI	52.2%	NI	NI	–	BG	–	–	NFN	–	BG	–	–

NI, not included; ND, not detected; OTR, out of target region; TL, tolerated; DT, deleterious; BG, benign; PD, Probably Damaging; PS, Possibly damaging; U, Unknown, NL, normal; DC, Disease causing; PM, polymorphism; NFN, non-functional neutral; NFL, non-functional low; FCM, functional medium; DG, Damaging; HF, harmful. *Approved for cancer types different from CUP.

**TABLE 2:** Mutation report of tumor, ccfDNA and CTCs from Pt#95.

				**SureSelect 92-gene panel (Agilent Technologies)**	**SureSelect 92-gene panel (Agilent Technologies)**	**Ampli1 OncoSeek and LowPass Kit (Menarini Silicon Biosystem)**	**Pathogenicity Prediction**								**Actional genes/drugs [OncoKB (Chakravarty et al., 2017)]**
**Gene**	**Position**	**Coding change**	**Amino acidic change**	**ccfDNA at diagnosis**	**Tumor FFPE**	**CTCs**	**SIFT** (Vaser et al., 2016)	**Polyphen2** (Adzhubei et al., 2010)	**LRT** (Chun and Fay, 2009)	**Mutation Taster** (Schwarz et al., 2014)	**Mutation Assessor** (Reva et al., 2011)	**FATHMM** (Shihab et al., 2013)	**CADD** (Kircher et al., 2014)	**VEST3** (Douville et al., 2016)	
ARID1A	chr1:26774679	c.4454_4457delTACA	p.I1485Rfs*19	32.6%	25%	NI	-	-	-	-	-	-	-	-	-
ARID1A	chr1:26774687	c.C4460T	p.A1487V	33.2%	27%	NI	TL	BG	DT	DC	NFN	TL	BG	HF	-
SPEN	chr1:15937527	c.G10391C	p.G3464A	31.0%	24%	NI	TL	BG	-	DC	NFL	TL	BG	BG	-
PIK3CG	chr7:106872869	c.G2218A	p.E740K	22.0%	8%	NI	TL	BG	NL	DC	NFL	TL	BG	BG	-
FGFR2	chr10:121515254	c.G814A	p.G272R	19.0%	NC	OTR	DT	PD	DT	DC	FCM	DG	HF	HF	Level 4/Debio1347, AZD4547, BGJ398, Erdafitinib
KMT2D	chr12:49031574	c.G13131A	p.W4377X	17.0%	FLQ	NI	DT	–	NL	DC^†^	–	–	HF	–	Level 3/no drug
FAT1	chr4:186621042	c.A5544C	p.Q1848H	13.1%	NC	NI	TL	PD	DT	DC	NFL	TL	BG	HF	–
KMT2C	chr7:152265180	c.G1042A	p.D348N	9.8%	FLQ	NI	TL	PD	U	DC	NFL	DG	BG	BG	–
KMT2C	chr7:152248140	c.A2294G	p.E765G	7.8%	ND	NI	TL	BG	DT	DC	NFL	DG	BG	BG	–
KMT2C	chr7:152235876	c.C2710T	p.R904X	7.5%	FLQ	NI	TL	–	NL	DC	–	–	HF	–	–
KMT2C	chr7:152235929	c.G2657A	p.R886H	6.8%	ND	NI	DT	PS	DT	DC	FCM	DG	HF	BG	–
KMT2C	chr7:152235861	c.A2725G	p.R909G	6.6%	ND	NI	TL	PD	DT	DC	FCM	DG	BG	HF	–
KMT2C	chr7:152248143	c.C2291T	p.S764F	4.9%	FLQ	NI	DT	BG	NL	DC	NFL	DG	BG	BG	–
KMT2C	chr7:152265209	c.C1013T	p.S338L	4.9%	ND	NI	TL	PD	U	DC	NFL	DG	BG	BG	–
KMT2C	chr7:152273774	c.G943A	p.G315S	4.9%	NC	NI	DT	PD	DT	DC	FCM	TL	HF	HF	–
KMT2C	chr7:152247966	c.T2468C	p.I823T	2.3%	NC	NI	TL	PD	DT	DC	NFL	DG	BG	HF	–
MGA	chr15:41767261	c.C8552T	p.P2851L	NC	5.20%	NI	TL	PS	NL	DC	NFN	DG	BG	BG	–
KMT2C	chr7:152163206	c.C10371A	p.D3457E	NC	6.30%	NI	TL	BG	NL	DC	NFL	DG	BG	BG	–
PTPRD	chr9:8521304	c.G904A	p.E302K	NC	5.00%	NI	–	PD	DT	DC	FCM	TL	HF	HF	–
ASXL2	chr2:25743628	c.G1929T	p.Q643H	NC	5.10%	NI	DT	PD	NL	DC	FCM	TL	BG	BG	–

*NI, not included; NC, not covered region; FLQ, filtered for low quality; ND, not detected; OTR, out of target region; TL, tolerated; DT, deleterious; BG, benign; PD, Probably Damaging; PS, Possibly damaging; U, Unknown, NL, normal; DC, Disease causing; NFN, non-functional neutral; NFL, non-functional low; FCM, functional medium; DG, Damaging; HF, harmful. ^†^Known to be disease causing.*

Single-cell genomic DNA from DEPArray NxT sorted cells, amplified using the *Ampli*1^TM^ WGA Kit, was processed to obtain Illumina-compatible libraries using the *Ampli*1^TM^ OncoSeek Panel and the *Ampli*1^TM^ LowPass kits. Conversely, tumor gDNA was directly processed with DEPArray^TM^ LibPrep and DEPArray^TM^ OncoSeek Panel kits. After sequencing on MiSeq platforms, raw data were analyzed using assay-specific applications on the MSBiosuite platform (Menarini Silicon Biosystems).

FFPE tumor tissue of Pt#71 was eligible for genetic testing using FoundationOne CDx (F1CDx) assay. F1CDx is performed exclusively as a laboratory service using DNA extracted from FFPE tumor samples. A library was prepared with a hybrid capture−based target enrichment approach and whole−genome shotgun library construction to detect substitutions, indels, copy number alterations, and selected rearrangements in a total of 324 genes. Sequencing was performed using the Illumina HiSeq 4000 platform, obtaining a >500 × median coverage. F1CDx specimens were also simultaneously profiled for tumor mutation burden (TMB) and microsatellite instability (MSI) status (further technical information is available at https://www.foundationmedicine.com/genomic-testing/foundation-one-cdx).

### Droplet Digital PCR Validation

Droplet Digital PCR technology (Bio-Rad, Hercules, CA, United States) along with Mutational and Copy Number Determination assays (Bio-Rad) were used to validate specific alterations in both genomic and ccfDNAs. A Copy Number Variation assay was used to assess the amplification of *FGFR2* gene (dHsaCB2500320, Bio-Rad) using *RPP30* as reference gene (dHsaCP2500350, Bio-Rad). A digestion with *Hae*III restriction enzyme was performed during ddPCR, according to the manufacturer’s instruction. Mutational assay for wild-type and mutated *ARID1A* (p.R1276^∗^) was performed with a custom IDT assay (02516372). For this assay, DNA digestion with *Hin*dIII restriction enzyme was performed only for gDNA. Probe-based Droplet Digital PCR experiment was performed according to the manufacturer’s instruction and thermal cycling conditions were 95°C for 10 min, 94°C for 30 s and 58°C for 1 min for 40 cycles, 98°C for 10 min, and an infinite hold at 4°C (ramping rate reduced to 2%). Droplet selection was performed individually for each well using QuantaSoft software v 1.7 (Bio-Rad) obtaining the final copy number variation or the wild-type/mutated allele fractions.

## Results

### Case Presentation

#### Pt#71

A 49-year-old woman with a history of primary biliary cholangitis (PBC) was admitted at Bologna University Hospital in June 2018 due to enlarged left cervical lymph nodes associated with severe asthenia, pruritus, and fever. A contrast-enhanced computed tomography (CT) scan showed multiple pathologic abdominal (epigastrium, mesogastrium, stomach curvature, hepatic hilum, pancreatic, and retroperitoneal regions) and left supraclavicular lymph nodes, confirmed also by a F-Fluorodeoxyglucose positron emission tomography/computed tomography (18-F-FDG PET/CT) scan. The patient underwent abdominal surgery leading to the excision of two lymph nodes. Pathological examination confirmed the presence of nodal metastasis from adenocarcinoma.

Immunohistochemistry staining reported positivity for keratin 7, keratin 20, and CDX2, and negativity for neuroendocrine markers (chromogranin, synaptophysin, and CD56). Therefore, the conclusive pathological diagnosis was suggestive of lymph node metastasis from adenocarcinoma of the gastrointestinal tract. Esophagogastroduodenoscopy (EGDS) and pancoloscopy (PC) failed to identify any neoplastic lesion. In addition, molecular analyses for the determination of MSI, HER2, ALK, and PD-L1 status (by IHC); *KRAS*, *NRAS*, *BRAF* mutational status and *MGMT* methylation status (by pyrosequencing); and *ROS1* and *MET* (by FISH) were all negative. Among the serum oncomarkers, the positive ones were Ca19.9 (291 U/ml, n.v < 40 U/ml), Ca125 (130.9 U/ml, n.v < 35 U/ml), and NSE (34 μg/L, n.v < 12 μg/L).

Taking into account the history of PBC and its association with a higher risk of cholangiocarcinoma, a magnetic resonance cholangiopancreatography (MRCP) and MRI with contrast was performed and ruled out the presence of any tumor lesion. Then, the patient was treated with FOLFOX-4 as first-line treatment every 2 weeks up to 11 courses, obtaining a partial response. A maintenance treatment based on De Gramont regimen (FULCV) was continued for four courses followed by a worsening of patient’s clinical condition and the appearance of pleural and abdominal effusions. Blood was collected for CTC analysis with CELLSEARCH and DEPArray at November 2018. Treatment with Pazopanib, a novel multi-kinase inhibitor (VEGFR1, VEGFR2, VEGFR3, PDGFR, FGFR, c-Kit, and c-Fms/CSF1R), was proposed due to the presence of *FGFR2* amplification (F1CDx testing). However, death occurred in June 2019 due to progressive disease and hepato-renal syndrome.

#### Pt#95

A 63-year-old woman was admitted at Bologna University Hospital in February 2019 due to an edema of the upper limb. Echo-Doppler scanning revealed thrombosis in basilica, axillary, subclavian, internal jugular, and external jugular veins. The patient experienced weight loss, asthenia, nausea, and cough.

A contrast-enhanced total body CT showed the presence of supra- and infradiaphragmatic enlarged lymph nodes associated with edema and diffuse suffusion of mesenteric and perivisceral tissues suggestive of peritoneal carcinosis, confirmed also by a 18F-FDG-PET/CT scan.

A CT-guided biopsy of a left paraortic lymph node led to a pathological diagnosis of node metastasis from poorly differentiated carcinoma with microglandular and signet-ring architecture. At immunohistochemistry, cells presented a focal positivity for K7 and CDX2 and negativity to K20 and synaptophysin. The immunophenotypical findings suggested to suspect a gastric primary tumor. Blood was collected for CTC analysis with CELLSEARCH and DEPArray and Parsortix at December 2019.

In May 2019, molecular analyses for the determination of MSI and HER2 status (by IHC) were negative, as well as *KRAS*, *NRAS*, *BRAF*, mutational status, and MGMT methylation status (by pyrosequencing). The patient underwent EGDS with ecoendoscopy, which found a suspicious area in the ampulla of Vater. Histological examination confirmed the presence of poorly differentiated adenocarcinoma with mixed, micro-glandular pattern, and signet-ring cells and a lymphangitic infiltration of the mucosa of duodenal type. IHC analysis reported a strong immunoreactivity to MUC1, MUC5AC, MUC6, and a complete negativity to MUC2 in addition to the previously identified K7^+^/K20^–^/CDX2^+^ profile, suggesting a likely bilio-pancreatic origin.

Starting from May 2019, the patient was treated with FOLFOX-4 regimen up to nine cycles, obtaining an initial partial response. In October 2019, the patient was admitted at the Emergency room of the Bologna University Hospital due to dyspnea and bilateral pleural effusion. The patient underwent pleural drainage and cytological examination, followed by video-assisted thoracoscopy and pleurodesis. The cell block was tested by IHC and the identified tumor cells were found to be reactive for BEREP4, K7, and CDX2 and negative for TTF1 and calretinin. Molecular analysis on pleural-derived cytological specimen was performed in the diagnostic setting. The analysis reported a pathogenic alteration in *FGFR2* gene (c.1150G > A, p. Gly384Arg, AF:33.4%); no gene amplification or fusion was detected. In December 2019, the patient started weekly gemcitabine regimen as second-line treatment. After 2 weeks from the first administration, the patient had uncontrolled ascitic effusion. Abdominal CT with and without contrast showed progressive disease due to evident radiological signs of peritoneal carcinosis. The patient died in January 2020 due to further progressive disease.

### Identification and Characterization of CTCs From CUP Patients Using CELLSEARCH and DEPArray or Parsortix Systems

CTCs from patients’ peripheral blood were isolated by combining CELLSEARCH and the DEPArray system. For Pt#71, 66 (CK^+^/CD45^–^/DAPI^+^) cells (none of which was positive for PD-L1) were captured with immunomagnetic beads. The isolated cells were then loaded on DEPArray that retrieved *n* = 12 CTCs. Three CTCs and one leukocyte as control were subjected to single-cell whole-genome amplification and sequencing.

**FIGURE 1:**
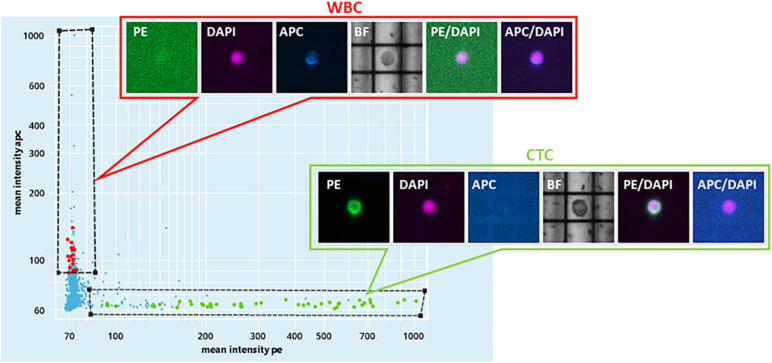
DEPArray NxT analysis. Scatter plot of the enriched blood sample of patient#95. On the *X*-axis, the mean intensity fluorescence of PE channel is reported, corresponding to the intensity of CK IF staining. On the *Y*-axis, the mean intensity of CD45-APC is represented.

For Pt#95, CELLSEARCH captured 133 CTCs cells (defined as above) from which DEPArray recovered 34 putative CTCs (Figure 1 and Supplementary Figure 1). A total number of 32 CTCs and four leukocytes ware subjected to single-cell whole-genome amplification, and 10 CTCs passed quality control criteria for low-pass whole-genome analysis.

**FIGURE 2:**
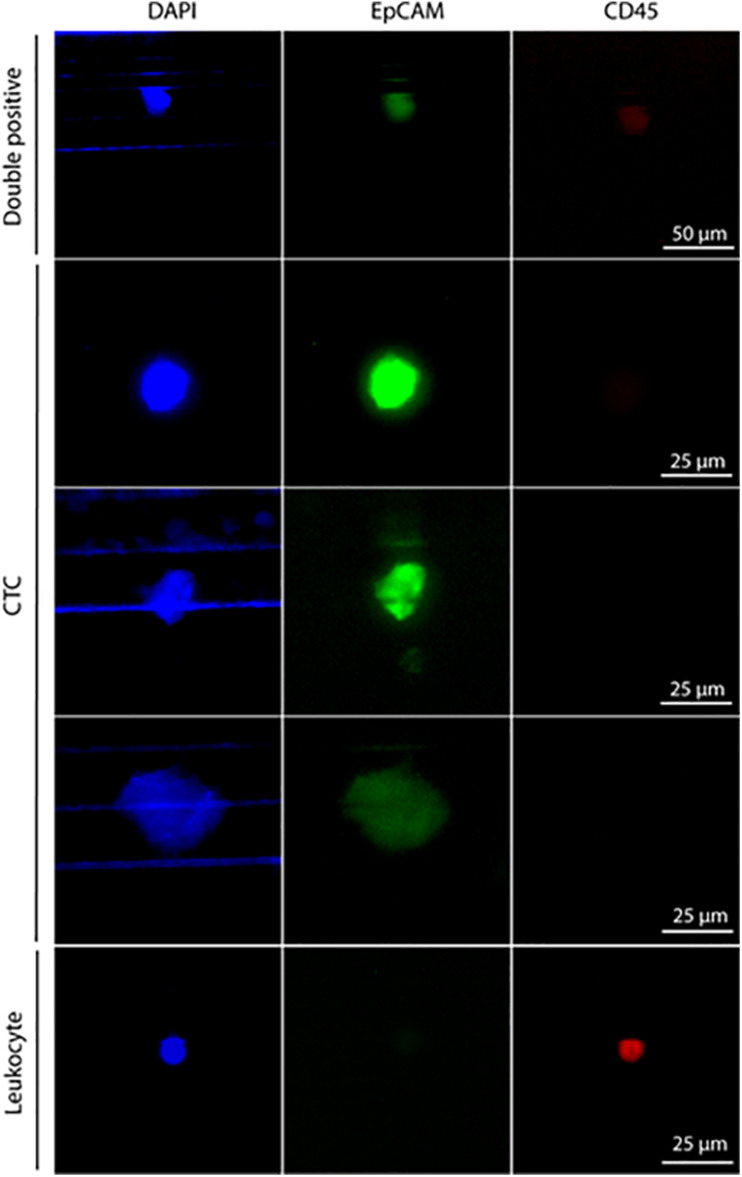
Staining of cells isolated with Parsortix from patient #95. Cells were forced to pass through a 6.5-μm cassette in the Parsortix system and then stained with different antibodies. Nuclei stained with DAPI (blue), CD45 (red), and EpCAM (green). From top to bottom: double-positive cells (CD45^+^, EpCAM^+^) of unknown significance, three exemplary CTCs (CD45^–^, EpCAM^+^), one exemplary leucocyte (CD45^+^, EpCAM^–^) Scale bar, 50 μm (double-positive cells) and 25 μm (CTCs and leucocyte).

Pt#95 whole blood was also used to isolate CTCs with the Parsortix system (ANGLE plc). Large cells were isolated with a 6.5 μm Parsortix cassette. Some EpCAM-positive and CD45-negative CTCs were captured in the cassette (Figure 2), together with EpCAM-positive and CD45-positive cells of unknown significance (Figure 2).

### Mutational Analysis of CTCs, ctDNA, and Tumor

#### Pt#71

**FIGURE 3:**
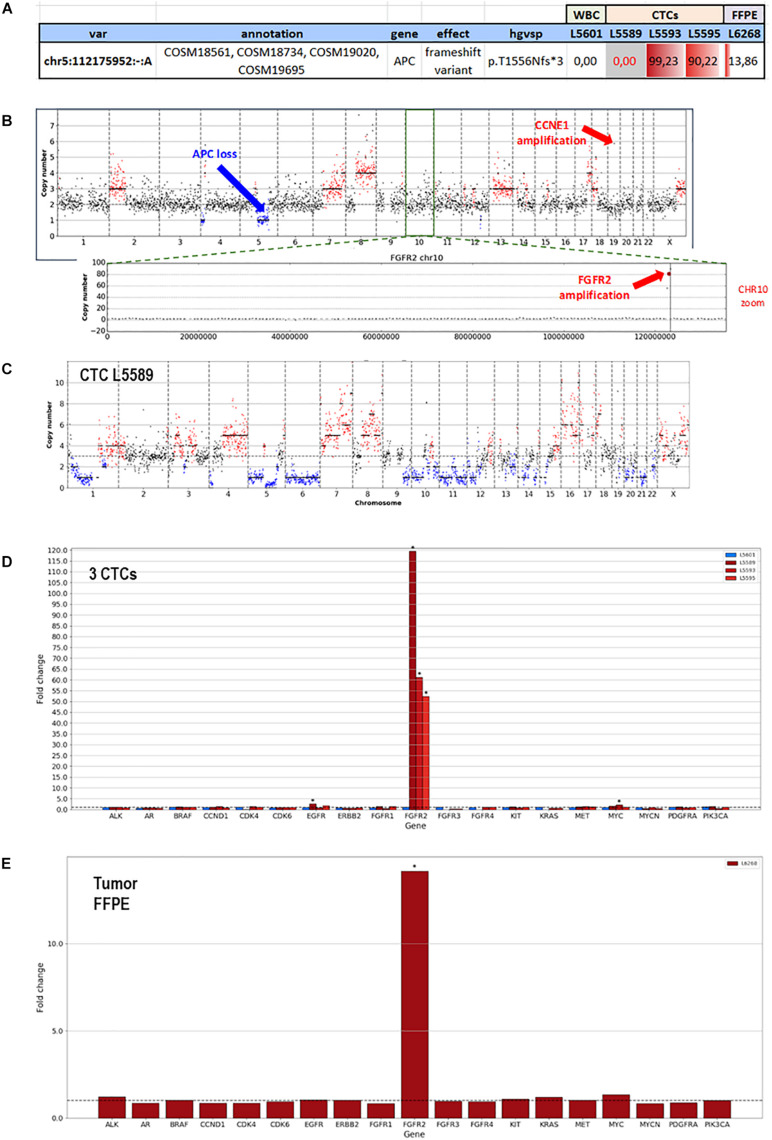
Whole-genome copy number profiles and mutation analysis of CTCs and FFPE gDNA from patient #71. **(A)** Detection of a homozygous somatic variant (absent in a single WBC), in two out of three CTCs in *APC* gene (APC:p.T1556Nfs*3), a region also subjected to LOH, and the detection of the same variant in the FFPE tissue at lower frequency, due to the possible contamination by normal cells. **(B)** Representative copy number profile of one CTC. Amplification signal was detected for *FGFR2* and *CCNE1* genes, indicated with red arrows. The LOH region in chromosome 5, comprising the *APC* gene, is indicated with a blue arrow. **(C)** LowPass profile of L5589 CTC showing the complete deletion of *APC* genomic region, in agreement with the lack of coverage in targeted sequencing (grayed out in **A**). **(D)** Copy number aberration (CNA) distribution showing *FGFR2* amplification in all three CTCs **(D)** and FFPE biopsy **(E)**.

F1CDx testing reported the amplification of *FGFR2* (Copy Number, CN: 49) and *CCNE1* (CN: 8) genes along with a somatic variant in *ARID1A* gene (p.R1276^∗^) with an allele frequency (AF) of 7.3%. Moreover, F1CDx reported an intermediate tumor mutational burden (13 mutations/Mb). Single-cell genome-wide characterization of CTCs (*Ampli*1 LowPass, Menarini Silicon Biosystems) allowed sub-chromosomal losses detection, including a LOH region comprising the *APC* gene, and pattern of extensive gains, including *FGFR2* and *CCNE1* genes (Figure 3).

The same alterations were confirmed on bulk tumor DNA. Moreover, targeted sequencing (*Ampli*1 OncoSeek panel, Menarini Silicon Biosystems) was able to detect the presence of *FGFR2* amplification in 3/3 CTCs and the inactivating homozygous somatic variant in *APC* gene (p.T1556Nfs^∗^3, AF: 90.2–99.2%). The APC somatic variant, which was missed by F1CDx, was detected (Figure 3) in two out of three analyzed CTCs, while the third CTC shows no coverage in the APC region (L5589, grayed out), in agreement with the deletion detected by the LowPass copy number profile in this cell, and a single-WBC control from the same sample (L5601) was wild type, as expected. The tumor gDNA target sequencing (DEPArray OncoSeek panel, Menarini Silicon Biosystems) confirmed the *FGFR2* amplification and reported the same frameshift variant in *APC*, even though with a lower allele frequency (p.T1556Nfs^∗^3, AF: 14%), likely due to contamination by normal cells. The single-copy homozygous variant in APC found in CTCs is coherent with a ∼15% variant frequency in the presence of 70% of normal cells in FFPE. Indeed, when setting the contamination parameter to 70% in the FFPE low-pass analysis, the bioinformatic pipeline was able to call, out of the increased noise, some CNA regions similar to those detected in CTCs, which were otherwise undetectable without the correction factor (Supplementary Figure 2).

The SureSelect 92-gene custom panel detected the APC insertion in bulk tumor DNA (AF:9.8%) and also in circulating cfDNAs analyzed at two different time points (ccfDNA at diagnosis, AF: 22.6% and ccfDNA at the progression of the disease, AF:29.9%). The tool *panelcn.MOPS* (Povysil et al., 2017) used to analyze target sequencing data confirmed the *FGFR2* and *CCNE1* gene amplification reporting a copy number alteration higher than four in both genes; however, no *APC* loss was detected. Moreover, we confirmed the mutation in *ARID1A* gene (p.R1276^∗^), *ERBB3* (p.S128R), *KEAP1* (p.R135H), *PALB2* (p.P807S), and *NTRK1* (p.Q736X), previously reported by F1CDx, both in the tumor and in the ccfDNAs. All detected variants and CNVs are listed in Tables 1, 3.

**TABLE 3:** Copy number (CN) alterations detected in Pt#71.

		**SureSelect 92-gene panel (Agilent Technologies)**	**SureSelect 92-gene panel (Agilent Technologies)**	**FoundatiOne CDx^TM^ (Roche)**	**Ampli1 OncoSeek and LowPass Kit (Menarini Silicon Biosystem)**	**DEPArray^TM^ OncoSeek and LowPass Kit (Menarini Silicon Biosystem)**
Gene	Alteration	Tumor FFPE (CN)	ccfDNAs (CN)	Tumor FFPE (CN)	CTC (CN)	Tumor FFPE (CN)
FGFR2	Amplification	>4	>4	49	> 80	82
CCNE1	Amplification	>4	>4	8	6	5
APC	Loss/deletion	Not detected	Not detected	Not detected	Detected	Detected

In Table 1, we reported other mutations detected by F1CDx in genes not included in the custom panel and some variants detected in ccfDNAs not observed in the tumor. Of note, we observed mutations in *ALK* (p.K434R, AF:7%), *EPHA5* (p.T473P, AF: 8.3%), *PTPRD* (p.Q1039H, AF:6.8%), and other alterations at lower frequency.

**FIGURE 4:**
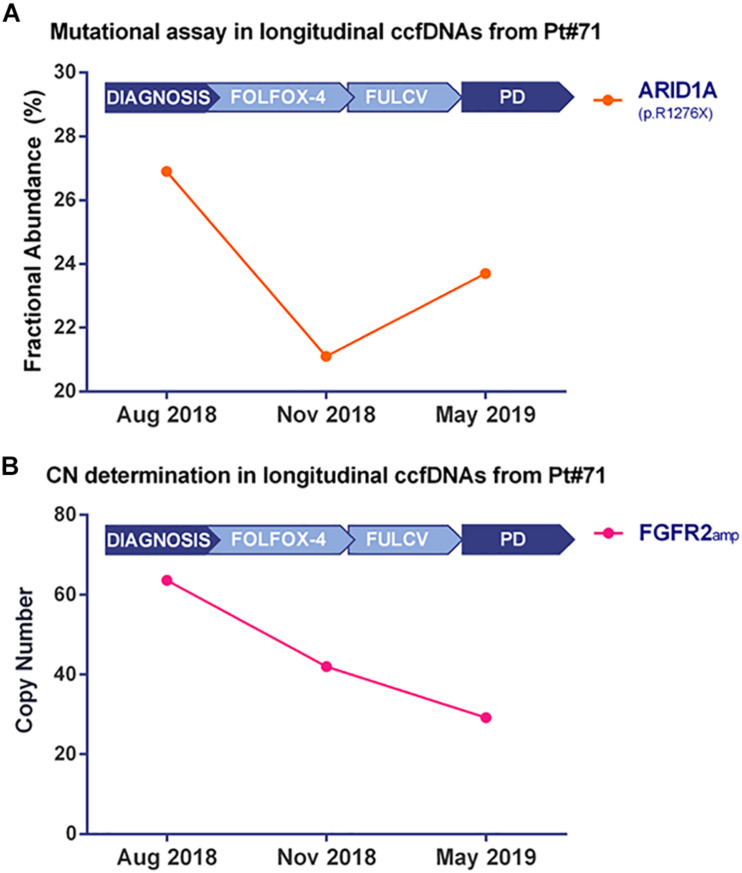
Kinetics of mutated fractional abundance and CNV in longitudinal ccfDNAs measured by Droplet Digital PCR. The graph in **(A)** illustrates *ARID1A* mutated fractional abundance (p.R1276X) during patient’s follow-up. This value decreases during treatment and later rises at the worsening of patient’s conditions; on the contrary, when monitoring *FGFR2* amplification at the same time points, as reported in **(B)**, a decreasing trend was detected from the diagnosis to disease progression.

Droplet Digital PCR technology validated the *ARID1A* mutation (Figure 4A and Supplementary Figure 3A) and the *FGFR2* amplification (Figure 4B and Supplementary Figure 3B) in bulk tumor DNA and in longitudinally collected ccfDNAs at three different time points of disease evolution. Of note, the frequency of *ARID1A* mutation decreases at time point B, during FOLFOX-4 treatment, then it increases during the progression of the disease, while the *FGFR2* amplification has a decreasing trend in terms of copy number value during disease progression. The Variant Allele Frequency reported by target sequencing at two time points was found to be consistent with the Fractional Abundance (FA% = mutated copies/mutated copies + wild-type copies) measured by ddPCR.

#### Pt#95

**FIGURE 5:**
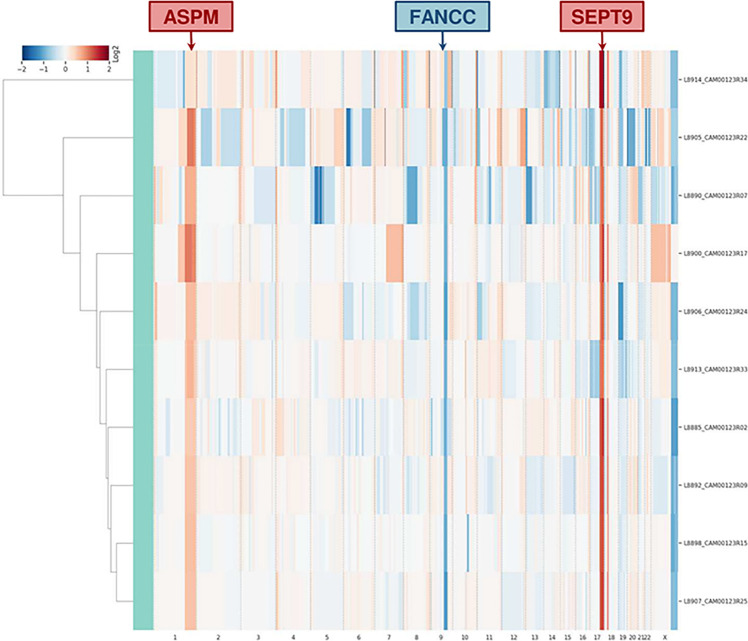
Clustering analysis of 10 CTCs based on whole-genome copy number profile. The heatmap represents the relative copy number profiles of 10 different CTCs isolated through CELLSEARCH^®^ and DEPArray^TM^ technologies. Segments of gains or deletions are color-coded according their relative log2 copy number ratios.

Single-cell whole-genome characterization of 10 CTCs detected a recurrent amplification signal in the regions of *ASPM* and *SEPT9* genes and a deletion including *FANCC* gene in all analyzed CTCs (Supplementary Figure 4), as shown in the CNV profile clustering in Figure 5. CTCs and bulk tumor DNA-targeted sequencing (*Ampli*1 OncoSeek Panel, Menarini Silicon Biosystems) reported no somatic mutation in the regions covered by the panel.

Target sequencing performed on ccfDNA and tumor FFPE tissue with the 92-gene custom panel identified 16 non-synonymous, somatic genetic alterations not detected in the normal cells in the ccfDNA and 8 in the tumor DNA (Table 2), including *ARID1A* deletion (I1485Rfs^∗^19, AF: 32.6%) and mutation (p. A1487V, AF: 33.2%) and a mutation in *FGFR2* gene (p.G272R, AF:19%). Of note, mutational analysis performed in the diagnostic setting (obtained with Oncomine Comprehensive Assay by Thermo Fisher Scientific) reported the same mutation in *FGFR2* gene; no amplifications or deletions were detected with Agilent custom panel, but regions including *ASPM*, *SEPT9*, and *FANCC* genes were not captured.

## Discussion

The identification of driver and actionable genetic alterations is crucial to broaden the treatment choices of cancer patients. This is particularly relevant for patients with CUP, who have reduced access to targeted and immunotherapies. One main reason resides in treatment protocols being designed in a tumor-gnostic way. Furthermore, CUPs are characterized by scarce availability of tumor material for genetic testing, due to the extensive use of tissue slices in immune-phenotypic testing required for diagnostic procedures.

In this pilot study, we took advantage of liquid biopsy approaches to genetically characterize CUP tumors using both CTCs and DNA as a source of tumor DNA, with the aim to identify druggable genetic alterations.

The isolation and counting of CTCs is becoming an important tool to evaluate aggressiveness and prognosis of different solid tumors. The CELLSEARCH system (Menarini Silicon Biosystems) is to date the only platform cleared by the FDA for detecting and enumerating CTCs in metastatic breast, prostate, and colorectal cancer patients, while other technologies are at preclinical and clinical stages of development. CTC count as measured by CELLSEARCH System proved to have prognostic value in different metastatic solid tumors, including breast (Cristofanilli et al., 2004), colorectal (Cohen et al., 2008), and gastric cancers (Matsusaka et al., 2010). In addition, the detection of one or more CTCs using this system was found to predict early recurrence and a worse prognosis in chemo naïve patients with non-metastatic breast cancer (Lucci et al., 2012). A considerable number of CTCs were detected in the two CUP patients enrolled in our study, and they were successfully enriched and enumerated by CELLSEARCH and isolated with DEPArray for downstream molecular analysis (*Ampli*1 kit).

Recently, CTC isolation methods based on label-free systems were also developed. In one patient, we used the Parsortix system (ANGLE plc) for the size-based enrichment of CTCs from peripheral blood of CUP patients. This approach ensured a size-based selection of CTCs without the need of specific markers, which is a potential issue when isolating these cells from CUP patients. Some CTCs enriched with the Parsortix system proved to be positive for EpCAM marker, therefore confirming their epithelial origin, although no genetic confirmation could be produced. We also detected cells that co-express hemato-epithelial markers, as previously reported for a subpopulation of epithelial ovarian cancer tumor cells (Akhter et al., 2018). In this paper, the authors proposed a cell–cell fusion theory to explain the origin of these peculiar cell population. Indeed, EpCAM^+^ and CD45^+^ cells show more invasive mesenchymal properties and overexpress genes related to drug resistance, anti-apoptosis, and stemness. The detection of cells with these characteristics in circulation could be associated with CUP highly invasive phenotype and deserves further investigations.

CTCs and ccfDNA were used for CUP genetic testing. Our results from two longitudinally monitored patients suggested that CTCs and ctDNA can be isolated from the blood of CUP patients and are suitable for genetic characterization. FFPE tumor tissue was used to validate the liquid biopsy results. In addition, we analyzed and compared different targeted sequencing approaches applied to tumor FFPE tissue, CTCs, and ccfDNA: FoundationOne CDx (Roche Foundation Medicine), OncoSeek Panels (*Ampli*1 and DEPArray OncoSeek Panels, Menarini Silicon Biosystems), and an in-house developed, Agilent SureSelect panel, focusing on 92 genes frequently mutated in CUPs and metastatic solid tumors. The comprehensive genomic analysis was highly overlapping among the different approaches, when the region was covered by all panels. Clearly, these three different technologies cover a different number of genes and hot spot regions, which explains the differential detection of some variants.

Interestingly, both CUP patients presented actionable *FGFR2* aberrations. Specifically, a ∼80-fold copy number amplification (Pt#71) and a gene mutation (Pt#95), both at the tumor and CTC/ctDNA level, suggest a potential implication of this pathway in tumor progression and aggressiveness (Dienstmann et al., 2014). FGFR2 is a member of the fibroblast growth factor receptor family, whose signaling pathway activates downstream effectors that play a crucial role in cell proliferation, survival, and migration (Turner and Grose, 2010). *FGFR2* rearrangements have been detected in 10–15% of intrahepatic cholangiocarcinomas (Arai et al., 2014; Silverman et al., 2019), amplifications in up to 15% of gastric cancers (Deng et al., 2012; Seo et al., 2017) and point mutations in about 10% of endometrial cancers (Byron et al., 2012); moreover, *FGFR2* amplification has also a prognostic significance in gastric cancers (Chang et al., 2014; Seo et al., 2017). Of note, in longitudinal ccfDNAs from Pt#71, we observed a progressive reduction of *FGFR2* CN during treatments, which mirrors the initial partial response. Taking into account the chemo-naïve status of the two patients and the unavailability of clinical trials testing multi-kinase or FGFR2 inhibitors in CUP patients, the patients were treated with platinum salts and fluoropyrimidines, as suggested by the current guidelines.

Pt#71 also presented an amplification in *CCNE1* (Cyclin E1) gene, which is known to be associated with poor prognosis in ovarian and triple-negative breast cancer, probably involved in the emergence of resistance to chemotherapy (Nakayama et al., 2010; Huang et al., 2020).

Another common genetic feature of the two tested CUP tumors was the presence of alterations in *ARID1A* gene, which is a tumor suppressor gene encoding the DNA-binding subunit of the chromatin remodeler complex SWI/SNF (Trizzino et al., 2018). Despite not being druggable, this gene is the most frequently mutated chromatin regulator across all human cancers (Kadoch et al., 2013) and its loss impairs RNA polymerase II (RNAPII) pausing. Compromised RNAPII pausing results in transcriptional dysregulation of active genes, such as *TP53* and *ESR1*, which could explain the oncogenic effects of ARID1A loss (Trizzino et al., 2018). The recurrence of these mutation in both reported cases open an interesting prospective on the role of ARID1A in regulating the aggressiveness of CUPs. Of note, when longitudinally evaluated in ccfDNAs of Pt#71, the Fractional Abundance of *ARID1A* mutation (p.R1276^∗^) decreased during treatment and then increases at disease progression, suggesting its potential involvement in disease worsening. Moreover, we noticed that in ccfDNAs at diagnosis and disease progression, in which the Fractional Abundance of *ARID1A*_*mut*_ was high, there was also the emergence of novel mutations in *TP53, ALK, EPHA5, FAT1*, and *MGA* genes, not detected in the tumor biopsy. *In vitro* experiments demonstrated that *ARID1A* deficiency leads to DNA replication stress (Tsai et al., 2020) and impaired checkpoint activation and repair of DNA double-strand breaks (DSBs), which sensitizes to DSB-inducing treatments (Shen et al., 2015). Moreover, ARID1A normally recruits MSH2 to chromatin during DNA replication and promotes mismatch repair (MMR); however, its deficiency is associated with the impairment of MMR and thus an increase in mutagenesis and mutational load (Shen et al., 2018). As a consequence, ARID1A deficiency has been investigated as a biomarker of response to anti-PD-1/PD-L1 immunotherapy treatment (Okamura et al., 2020); alterations in this gene were found to be associated with a longer PFS in patients treated with anti-PD-1/PD-L1 immunotherapy (regardless of microsatellite and tumor mutational burden status). It has been demonstrated that 15% of CUP patients harbor alterations in *ARID1A* gene (MSK impact study; Zehir et al., 2017). Therefore, immune checkpoint inhibitors could be considered as a therapeutic option for ARID1A mutated CUP cases. In the two CUP cases investigated in this study, ARID1A-dependent accumulation of genetic alterations could explain the number of novel mutations that were longitudinally detected in the ccfDNA.

However, another explanation could be that the mutation load was boosted by the mutagenic activity of oxaliplatin (L-OHP), a DNA-alkylating-like agent, which is included in FOLFOX-4 chemotherapy combination along with Leucovorin and 5-fluorouracil (5-FU) (Silva et al., 2005; Kawamoto et al., 2012). This chemotherapy regimen is commonly used to manage metastatic colorectal cancer (Benson et al., 2014), but also administered to CUP patients with K20^+^/K7^–^/CDX2^+^ IHC signature.

Overall, our analysis constitutes the proof of concept needed to assess the relevance of liquid biopsy genetic testing in metastatic cancer of unknown origin and underlies the importance of using dedicated NGS panels to identify actionable genetic alterations.

Altogether, our results support the use of liquid biopsy, either using CTCs or ccfDNA, in the genetic characterization of CUPs and identification of driver mutations, with the advantage of a minimally invasive approach and potential longitudinal monitoring. This is a proof-of-concept study on a small number of patients, but given the rarity of the disease and the great need for effective therapies, we believe we provided valuable novel data. We can also conclude that a timely genetic testing and the use of broad or CUP-dedicated NGS panels, covering the most frequently mutated genes, are of the outmost importance to offer these patients novel therapeutic options and potentially improve their survival.

## Data Availability Statement

The datasets presented in this article are not readily available because patients have consented to the use of their individual genetic data for biomedical research, but not for unlimited public data release. Requests to access the datasets should be directed to corresponding author.

## Ethics Statement

The studies involving human participants were reviewed and approved by the Ethics Committee Center Emilia-Romagna Region – Italy (protocol 130/2016/U/Tess). The patients/participants provided their written informed consent to participate in this study.

## Author Contributions

NL, IS, EP, MR, MG, PT, and SV carried out the experiments and data analysis. MF wrote the manuscript with support from NL, IS, FG, AA, FF, NM, MP, and SS. AD’E and AA helped supervise the project. MF conceived the original idea and supervised the project. All authors contributed to the article and approved the submitted version.

## Conflict of Interest

MG, PT, FF, and NM were employees of Menarini Silicon Biosystems and co-inventors in patents assigned to the company, regarding certain company products used in this study. AA received honoraria (self) for advisory board participation: BMS, MSD, ROCHE, Astra Zeneca, and Eli-Lilly Research Grants to my Institution: Celgene, BMS, Ipsen, and Roche. The remaining authors declare that the research was conducted in the absence of any commercial or financial relationships that could be construed as a potential conflict of interest.

## Footnotes

1 https://earray.chem.agilent.com/suredesign/

## Supplementary Material

Supplementary Figure 1 DEPArray NxT representative CTC images from patient #95. Cell gallery acquired by DEPArray, showing single fluorescent channels and overlays, and bright field (BF) images where the DEPArray electrodes (pitch 20μm) are visible.

Supplementary Figure 2 Patient #71: Low-Pass Whole Genome CNA profiles. **(A)** FFPE without compensation: the signal produced by normal cells impact the copy-number alterations (CNA) call. **(B)** Pure single-CTC: clear CNA calls with digital copy-number values (e.g., Chr 8q *n* = 4). **(C)** FFPE analysis set at 70% normal DNA contamination: signal is amplified and, among increased noise, some true positive CNA detected also in CTCs are called (green arrows) along with putative false-positives (orange arrows).

Supplementary Figure 3 Mutational assay and copy number determination assay by Droplet Digital PCR. The graph reports ddPCR outputs of *ARID1A* mutated fractional abundance (p.R1276X) **(A)** and in *FGFR2* amplification **(B)** during patient’s follow-up at three different time points.

Supplementary Figure 4 Patient #95 single-CTCs images and CNA profiles. Cell gallery acquired by DEPArray, showing single fluorescent channels and overlays, and bright field (BF) images for 10 CTCs, paired with the corresponding CNA profile.

Supplementary Table 1 List of genes and regions included in SureSelect custom panel.

## References

[B1] Aceto N. Bardia A. Miyamoto D. T. Donaldson M. C. Wittner B. S. Spencer J. A. (2014). Circulating tumor cell clusters are oligoclonal precursors of breast cancer metastasis. *Cell* 158 1110–1122. 10.1016/j.cell.2014.07.013 25171411 PMC4149753

[B2] Adzhubei I. A. Schmidt S. Peshkin L. Ramensky V. E. Gerasimova A. Bork P. (2010). A method and server for predicting damaging missense mutations. *Nat Methods* 7 248–249. 10.1038/nmeth0410-248 20354512 PMC2855889

[B3] Akhter M. Z. Sharawat S. K. Kumar V. Kochat V. Equbal Z. Ramakrishnan M. (2018). Aggressive serous epithelial ovarian cancer is potentially propagated by EpCAM(+)CD45(+) phenotype. *Oncogene* 37 2089–2103. 10.1038/s41388-017-0106-y 29379166

[B4] Amintas S. Bedel A. Moreau-Gaudry F. Boutin J. Buscail L. Merlio J. P. (2020). Circulating tumor cell clusters: united we stand divided we fall. *Int. J. Mol. Sci.* 21:2653. 10.3390/ijms21072653 32290245 PMC7177734

[B5] Arai Y. Totoki Y. Hosoda F. Shirota T. Hama N. Nakamura H. (2014). Fibroblast growth factor receptor 2 tyrosine kinase fusions define a unique molecular subtype of cholangiocarcinoma. *Hepatology* 59 1427–1434. 10.1002/hep.26890 24122810

[B6] Barber L. J. Mansukhani S. Herman B. Arezi B. Gerlinger M. (2017). *Application Note: Ultra-sensitive Cancer Liquid Biopsy Analysis with the Agilent SureSelectXT HS Target Enrichment Workflow.* Santa Clara, CA: Agilent Technologies.

[B7] Benson A. B. III Venook A. P. Bekaii-Saab T. Chan E. Chen Y. J. Cooper H. S. (2014). Colon cancer, version 3.2014. *J. Natl. Compr. Canc. Netw.* 12 1028–1059. 10.6004/jnccn.2014.0099 24994923

[B8] Byron S. A. Gartside M. Powell M. A. Wellens C. L. Gao F. Mutch D. G. (2012). FGFR2 point mutations in 466 endometrioid endometrial tumors: relationship with MSI, KRAS, PIK3CA, CTNNB1 mutations and clinicopathological features. *PLoS One* 7:e30801. 10.1371/journal.pone.0030801 22383975 PMC3285611

[B9] Chakravarty D. Gao J. Phillips S. Kundra R. Zhang H. Wang J. (2017). OncoKB: a precision oncology knowledge base. *JCO Precis. Oncol.* 2017:PO.17.00011. 10.1200/po.17.00011 28890946 PMC5586540

[B10] Chang J. Liu X. Wang S. Zhang Z. Wu Z. Zhang X. (2014). Prognostic value of FGFR gene amplification in patients with different types of cancer: a systematic review and meta-analysis. *PLoS One* 9:e105524. 10.1371/journal.pone.0105524 25171497 PMC4149366

[B11] Chun S. Fay J. C. (2009). Identification of deleterious mutations within three human genomes. *Genome Res* 19 1553–1561. 10.1101/gr.092619.109 19602639 PMC2752137

[B12] Cinausero M. Laprovitera N. De Maglio G. Gerratana L. Riefolo M. Macerelli M. (2019). KRAS and ERBB-family genetic alterations affect response to PD-1 inhibitors in metastatic nonsquamous NSCLC. *Ther. Adv. Med. Oncol.* 11:1758835919885540. 10.1177/1758835919885540 31798692 PMC6859675

[B13] Cohen S. J. Punt C. J. Iannotti N. Saidman B. H. Sabbath K. D. Gabrail N. Y. (2008). Relationship of circulating tumor cells to tumor response, progression-free survival, and overall survival in patients with metastatic colorectal cancer. *J. Clin. Oncol.* 26 3213–3221. 10.1200/JCO.2007.15.8923 18591556

[B14] Conway A. M. Mitchell C. Cook N. (2019). Challenge of the unknown: how can we improve clinical outcomes in cancer of unknown primary? *J. Clin. Oncol.* 37 2089–2090. 10.1200/jco.19.00449 31211603

[B15] Cristofanilli M. Budd G. T. Ellis M. J. Stopeck A. Matera J. Miller M. C. (2004). Circulating tumor cells, disease progression, and survival in metastatic breast cancer. *N. Engl. J. Med.* 351 781–791. 10.1056/NEJMoa040766 15317891

[B16] Deng N. Goh L. K. Wang H. Das K. Tao J. Tan I. B. (2012). A comprehensive survey of genomic alterations in gastric cancer reveals systematic patterns of molecular exclusivity and co-occurrence among distinct therapeutic targets. *Gut* 61 673–684. 10.1136/gutjnl-2011-301839 22315472 PMC3322587

[B17] Dienstmann R. Rodon J. Prat A. Perez-Garcia J. Adamo B. Felip E. (2014). Genomic aberrations in the FGFR pathway: opportunities for targeted therapies in solid tumors. *Ann. Oncol.* 25 552–563. 10.1093/annonc/mdt419 24265351 PMC4433501

[B18] Douville C. Masica D. L. Stenson P. D. Cooper D. N. Gygax D. M. Kim R. (2016). Assessing the pathogenicity of insertion and deletion variants with the variant effect scoring tool (VEST-Indel). *Hum. Mutat.* 37 28–35. 10.1002/humu.22911 26442818 PMC5057310

[B19] Drapkin B. J. George J. Christensen C. L. Mino-Kenudson M. Dries R. Sundaresan T. (2018). Genomic and functional fidelity of small cell lung cancer patient-derived xenografts. *Cancer Discov.* 8 600–615. 10.1158/2159-8290.CD-17-0935 29483136 PMC6369413

[B20] Fizazi K. Greco F. A. Pavlidis N. Daugaard G. Oien K. Pentheroudakis G. (2015). Cancers of unknown primary site: ESMO clinical practice guidelines for diagnosis, treatment and follow-up. *Ann. Oncol.* 26 (Suppl. 5) v133–v138. 10.1093/annonc/mdv305 26314775

[B21] Haratani K. Hayashi H. Takahama T. Nakamura Y. Tomida S. Yoshida T. (2019). Clinical and immune profiling for cancer of unknown primary site. *J. Immunother. Cancer* 7:251. 10.1186/s40425-019-0720-z 31519206 PMC6743146

[B22] Huang X. Wu H. M. Zhu C. Shao D. Guo D. Zhou Y. (2020). Next generation sequencing reveals CCNE1 amplification as an independent prognostic factor for triple negative breast cancer (TNBC) patients. *J. Clin. Oncol.* 38:558. 10.1200/JCO.2020.38.15_suppl.55831821071

[B23] Ignatiadis M. Dawson S. J. (2014). Circulating tumor cells and circulating tumor DNA for precision medicine: dream or reality? *Ann. Oncol.* 25 2304–2313. 10.1093/annonc/mdu480 25336116

[B24] Joosse S. A. Gorges T. M. Pantel K. (2015). Biology, detection, and clinical implications of circulating tumor cells. *EMBO Mol. Med.* 7 1–11. 10.15252/emmm.201303698 25398926 PMC4309663

[B25] Kadoch C. Hargreaves D. C. Hodges C. Elias L. Ho L. Ranish J. (2013). Proteomic and bioinformatic analysis of mammalian SWI/SNF complexes identifies extensive roles in human malignancy. *Nat. Genet.* 45 592–601. 10.1038/ng.2628 23644491 PMC3667980

[B26] Karczewski K. J. Francioli L. C. Tiao G. Cummings B. B. Alfoldi J. Wang Q. (2020). The mutational constraint spectrum quantified from variation in 141,456 humans. *Nature* 581 434–443. 10.1038/s41586-020-2308-7 32461654 PMC7334197

[B27] Kato S. Krishnamurthy N. Banks K. C. De P. Williams K. Williams C. (2017). Utility of genomic analysis in circulating tumor DNA from patients with carcinoma of unknown primary. *Cancer Res.* 77 4238–4246. 10.1158/0008-5472.CAN-17-0628 28642281 PMC5729906

[B28] Kawamoto Y. Tsuchihara K. Yoshino T. Ogasawara N. Kojima M. Takahashi M. (2012). KRAS mutations in primary tumours and post-FOLFOX metastatic lesions in cases of colorectal cancer. *Br. J. Cancer* 107 340–344. 10.1038/bjc.2012.218 22617127 PMC3394966

[B29] Kircher M. Witten D. M. Jain P. O’Roak B. J. Cooper G. M. Shendure J. (2014). A general framework for estimating the relative pathogenicity of human genetic variants. *Nat. Genet.* 46 310–315. 10.1038/ng.2892 24487276 PMC3992975

[B30] Komine K. Inoue M. Otsuka K. Fukuda K. Nanjo H. Shibata H. (2014). Utility of measuring circulating tumor cell counts to assess the efficacy of treatment for carcinomas of unknown primary origin. *Anticancer Res.* 34 3165–3168. 24922689

[B31] Laprovitera N. Riefolo M. Ambrosini E. Klec C. Pichler M. Ferracin M. (2021). Cancer of unknown primary: challenges and progress in clinical management. *Cancers* 13:451. 10.3390/cancers13030451 33504059 PMC7866161

[B32] Lucci A. Hall C. S. Lodhi A. K. Bhattacharyya A. Anderson A. E. Xiao L. (2012). Circulating tumour cells in non-metastatic breast cancer: a prospective study. *Lancet Oncol.* 13 688–695. 10.1016/S1470-2045(12)70209-7 22677156

[B33] Matsusaka S. Chin K. Ogura M. Suenaga M. Shinozaki E. Mishima Y. (2010). Circulating tumor cells as a surrogate marker for determining response to chemotherapy in patients with advanced gastric cancer. *Cancer Sci.* 101 1067–1071. 10.1111/j.1349-7006.2010.01492.x 20219073 PMC11159155

[B34] Nakayama N. Nakayama K. Shamima Y. Ishikawa M. Katagiri A. Iida K. (2010). Gene amplification CCNE1 is related to poor survival and potential therapeutic target in ovarian cancer. *Cancer* 116 2621–2634. 10.1002/cncr.24987 20336784

[B35] Okamura R. Kato S. Lee S. Jimenez R. E. Sicklick J. K. Kurzrock R. (2020). ARID1A alterations function as a biomarker for longer progression-free survival after anti-PD-1/PD-L1 immunotherapy. *J. Immunother. Cancer.* 8:e000438. 10.1136/jitc-2019-000438 32111729 PMC7057434

[B36] Pairawan S. Hess K. R. Janku F. Sanchez N. S. Mills Shaw K. R. Eng C. (2020). Cell-free circulating tumor DNA variant allele frequency associates with survival in metastatic cancer. *Clin. Cancer Res.* 26 1924–1931. 10.1158/1078-0432.CCR-19-0306 31852833 PMC7771658

[B37] Povysil G. Tzika A. Vogt J. Haunschmid V. Messiaen L. Zschocke J. (2017). panelcn.MOPS: copy-number detection in targeted ngs panel data for clinical diagnostics. *Hum. Mutat.* 38 889–897. 10.1002/humu.23237 28449315 PMC5518446

[B38] Reva B. Antipin Y. Sander C. (2011). Predicting the functional impact of protein mutations: application to cancer genomics. *Nucleic Acids Res.* 39:e118. 10.1093/nar/gkr407 21727090 PMC3177186

[B39] Riethdorf S. Fritsche H. Muller V. Rau T. Schindlbeck C. Rack B. (2007). Detection of circulating tumor cells in peripheral blood of patients with metastatic breast cancer: a validation study of the cell search system. *Clin. Cancer Res.* 13 920–928. 10.1158/1078-0432.CCR-06-1695 17289886

[B40] Ross J. S. Wang K. Gay L. Otto G. A. White E. Iwanik K. (2015). Comprehensive genomic profiling of carcinoma of unknown primary site: new routes to targeted therapies. *JAMA Oncol.* 1 40–49. 10.1001/jamaoncol.2014.216 26182302

[B41] Schwarz J. M. Cooper D. N. Schuelke M. Seelow D. (2014). MutationTaster2: mutation prediction for the deep-sequencing age. *Nat. Methods* 11 361–362. 10.1038/nmeth.2890 24681721

[B42] Seo S. Park S. J. Ryu M. H. Park S. R. Ryoo B. Y. Park Y. S. (2017). Prognostic impact of fibroblast growth factor receptor 2 gene amplification in patients receiving fluoropyrimidine and platinum chemotherapy for metastatic and locally advanced unresectable gastric cancers. *Oncotarget* 8 33844–33854. 10.18632/oncotarget.12953 27802183 PMC5464916

[B43] Shen J. Ju Z. Zhao W. Wang L. Peng Y. Ge Z. (2018). ARID1A deficiency promotes mutability and potentiates therapeutic antitumor immunity unleashed by immune checkpoint blockade. *Nat. Med.* 24 556–562. 10.1038/s41591-018-0012-z 29736026 PMC6076433

[B44] Shen J. Peng Y. Wei L. Zhang W. Yang L. Lan L. (2015). ARID1A deficiency impairs the dna damage checkpoint and sensitizes cells to parp inhibitors. *Cancer Discov.* 5 752–767. 10.1158/2159-8290.cd-14-0849 26069190 PMC4497871

[B45] Shihab H. A. Gough J. Cooper D. N. Stenson P. D. Barker G. L. Edwards K. J. I (2013). Predicting the functional, molecular, and phenotypic consequences of amino acid substitutions using hidden Markov models. *Hum. Mutat.* 34 57–65. 10.1002/humu.22225 23033316 PMC3558800

[B46] Silva M. J. Costa P. Dias A. Valente M. Louro H. Boavida M. G. (2005). Comparative analysis of the mutagenic activity of oxaliplatin and cisplatin in the Hprt gene of CHO cells. *Environ. Mol. Mutagen.* 46 104–115. 10.1002/em.20138 15887215

[B47] Silverman I. M. Murugesan K. Lihou C. F. Féliz L. Frampton G. M. Newton R. C. (2019). Comprehensive genomic profiling in FIGHT-202 reveals the landscape of actionable alterations in advanced cholangiocarcinoma. *J. Clin. Oncol.* 37 (15 Suppl.):4080. 10.1200/JCO.2019.37.15_suppl.4080

[B48] Sparano J. O’Neill A. Alpaugh K. Wolff A. C. Northfelt D. W. Dang C. T. (2018). Association of circulating tumor cells with late recurrence of estrogen receptor-positive breast cancer: a secondary analysis of a randomized clinical trial. *JAMA Oncol.* 4 1700–1706. 10.1001/jamaoncol.2018.2574 30054636 PMC6385891

[B49] Tanizaki J. Y. Akiyoshi K. Minami K. Ueda H. Takiguchi H. Nakayama Kondoh H. (2020). NivoCUP: an open-label phase II study on the efficacy of nivolumab in cancer of unknown primary. *J. Clin. Oncol.* 38 (15 Suppl.):106. 10.1200/JCO.2020.38.15_suppl.10631675249

[B50] Trizzino M. Barbieri E. Petracovici A. Wu S. Welsh S. A. Owens T. A. (2018). The tumor suppressor arid1a controls global transcription via pausing of RNA Polymerase II. *Cell Rep.* 23 3933–3945. 10.1016/j.celrep.2018.05.097 29949775 PMC6146183

[B51] Tsai S. Chang E. Y.-c. Fournier L.-A. Wells J. P. Minaker S. W. Zhu Y. D. (2020). ARID1A regulates R-loop associated DNA replication stress. *bioRxiv* [Preprint]. 10.1101/2020.11.16.384446 PMC8055027 33826602

[B52] Turner N. Grose R. (2010). Fibroblast growth factor signalling: from development to cancer. *Nat. Rev. Cancer* 10 116–129. 10.1038/nrc2780 20094046

[B53] Varadhachary G. R. Raber M. N. (2014). Cancer of unknown primary site. *N. Engl. J. Med.* 371 757–765. 10.1056/NEJMra1303917 25140961

[B54] Varghese A. M. Arora A. Capanu M. Camacho N. Won H. H. Zehir A. (2017). Clinical and molecular characterization of patients with cancer of unknown primary in the modern era. *Ann. Oncol.* 28 3015–3021. 10.1093/annonc/mdx545 29045506 PMC5834064

[B55] Vaser R. Adusumalli S. Leng S. N. Sikic M. Ng P. C. (2016). SIFT missense predictions for genomes. *Nat. Protoc.* 11 1–9. 10.1038/nprot.2015.123 26633127

[B56] Wang K. Li M. Hakonarson H. (2010). ANNOVAR: functional annotation of genetic variants from high-throughput sequencing data. *Nucleic Acids Res.* 38:e164. 10.1093/nar/gkq603 20601685 PMC2938201

[B57] Yu M. Bardia A. Aceto N. Bersani F. Madden M. W. Donaldson M. C. (2014). Cancer therapy. ex vivo culture of circulating breast tumor cells for individualized testing of drug susceptibility. *Science* 345 216–220. 10.1126/science.1253533 25013076 PMC4358808

[B58] Yu M. Stott S. Toner M. Maheswaran S. Haber D. A. (2011). Circulating tumor cells: approaches to isolation and characterization. *J. Cell Biol.* 192 373–382. 10.1083/jcb.201010021 21300848 PMC3101098

[B59] Zehir A. Benayed R. Shah R. H. Syed A. Middha S. Kim H. R. (2017). Mutational landscape of metastatic cancer revealed from prospective clinical sequencing of 10,000 patients. *Nat. Med.* 23 703–713. 10.1038/nm.4333 28481359 PMC5461196

[B60] Zhao P. Zhou W. Liu C. Zhang H. Cheng Z. Wu W. (2019). Establishment and characterization of a CTC cell line from peripheral blood of breast cancer patient. *J. Cancer* 10 6095–6104. 10.7150/jca.33157 31762819 PMC6856591

