# Supplementary figures and images for: Genetic Characterization of Cancer of Unknown Primary Using Liquid Biopsy Approaches
Source: Front Cell Dev Biol. 2021 Jun 10;9:666156. doi: 10.3389/fcell.2021.666156 (PMC8222689; doi:10.3389/fcell.2021.666156)

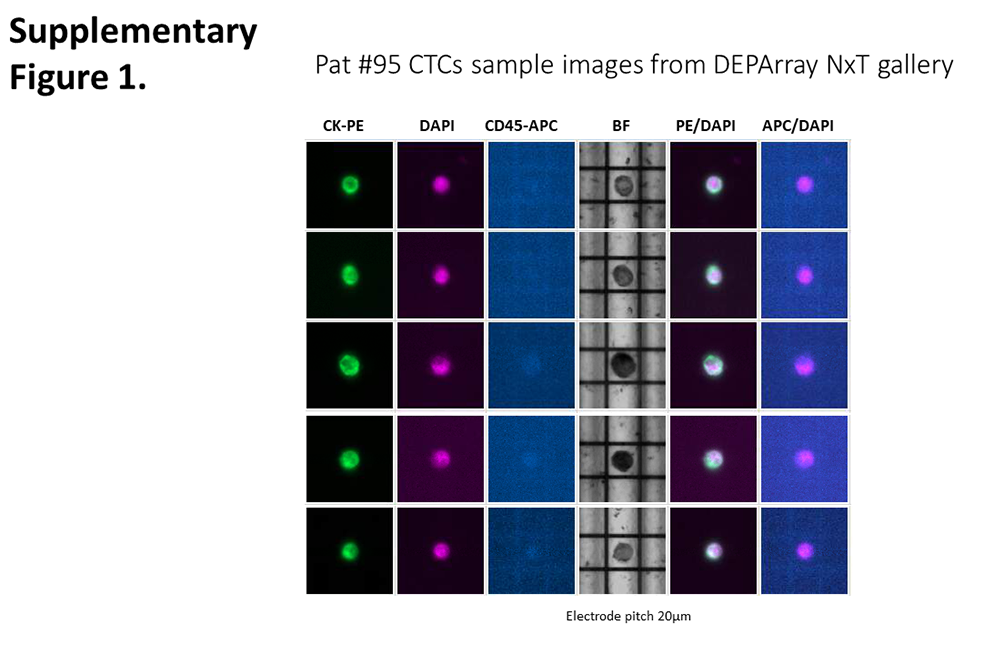

Supplement: Supplementary Figure 1 — DEPArray NxT representative CTC images from patient #95. Cell gallery acquired by DEPArray, showing single fluorescent channels and overlays, and bright field (BF) images where the DEPArray electrodes (pitch 20μm) are visible. [file Image_1.TIF]

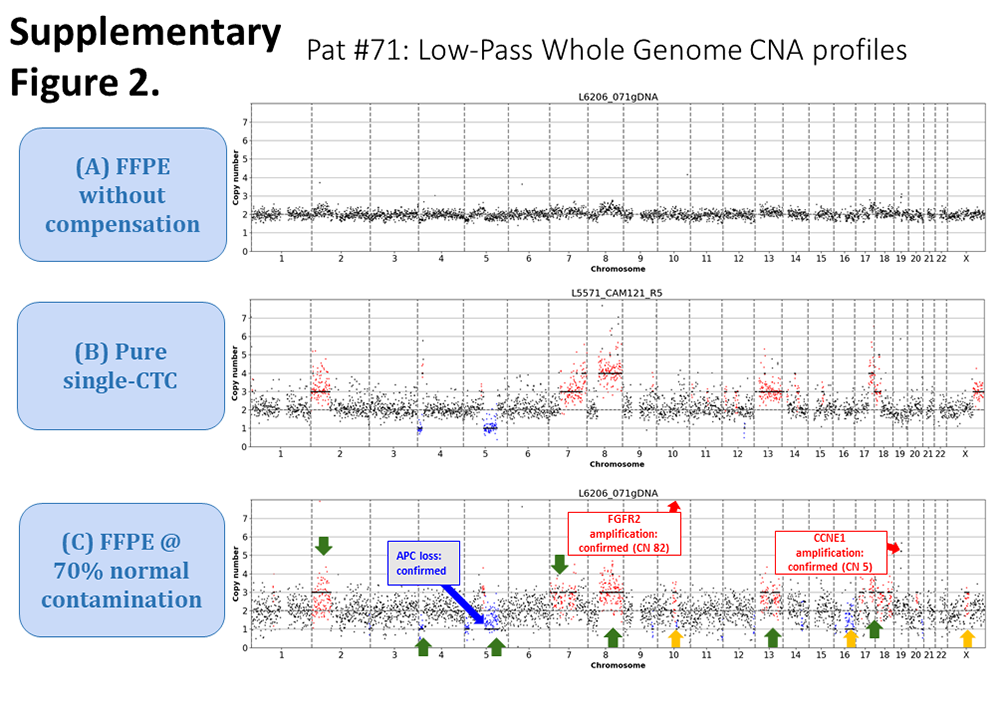

Supplement: Supplementary Figure 2 — Patient #71: Low-Pass Whole Genome CNA profiles. (A) FFPE without compensation: the signal produced by normal cells impact the copy-number alterations (CNA) call. (B) Pure single-CTC: clear CNA calls with digital copy-number values (e.g., Chr 8q n = 4). (C) FFPE analysis set at 70% normal DNA contamination: signal is amplified and, among increased noise, some true positive CNA detected also in CTCs are called (green arrows) along with putative false-positives (orange arrows). [file Image_2.TIF]

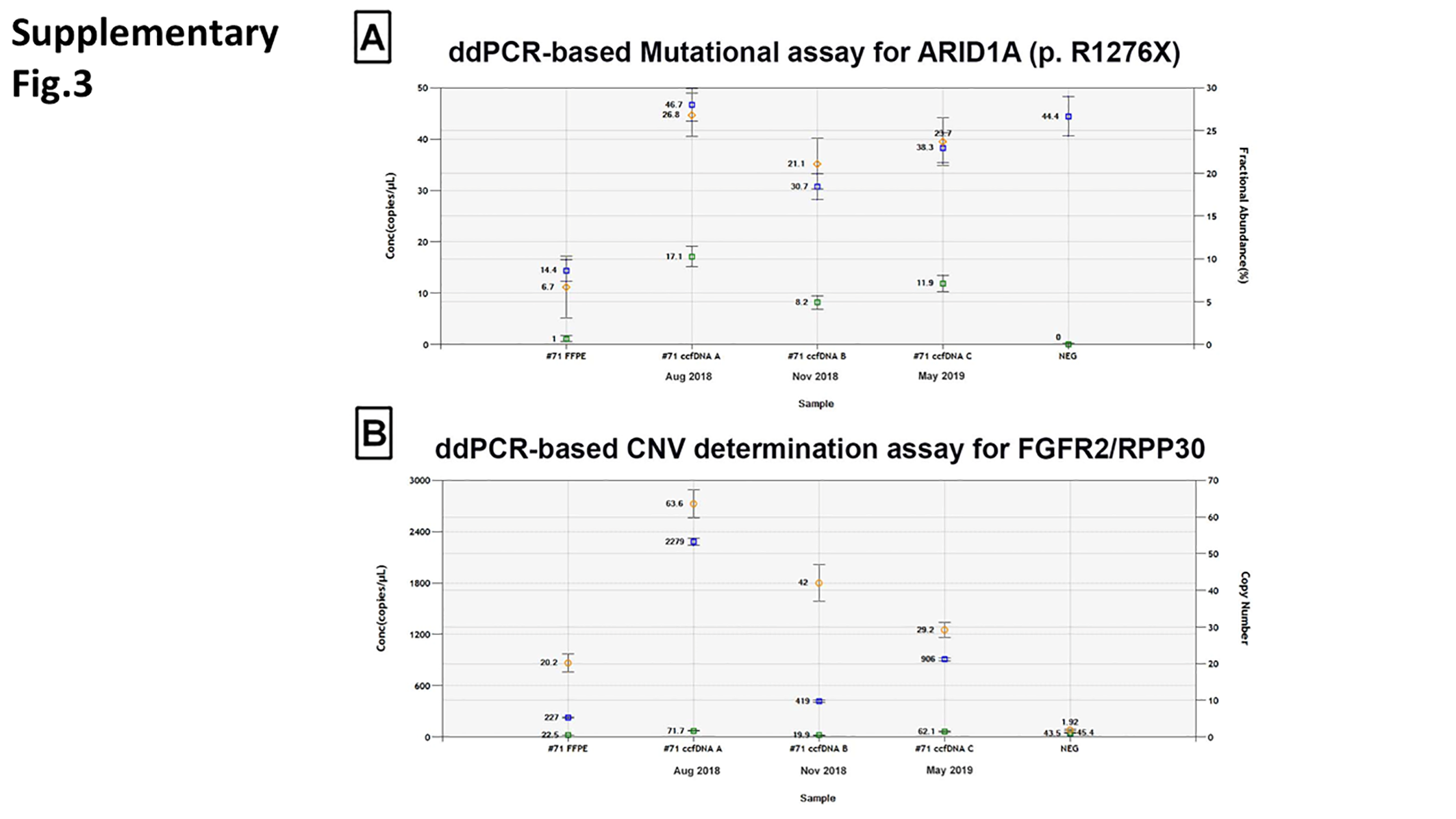

Supplement: Supplementary Figure 3 — Mutational assay and copy number determination assay by Droplet Digital PCR. The graph reports ddPCR outputs of ARID1A mutated fractional abundance (p.R1276X) (A) and in FGFR2 amplification (B) during patient’s follow-up at three different time points. [file Image_3.TIF]

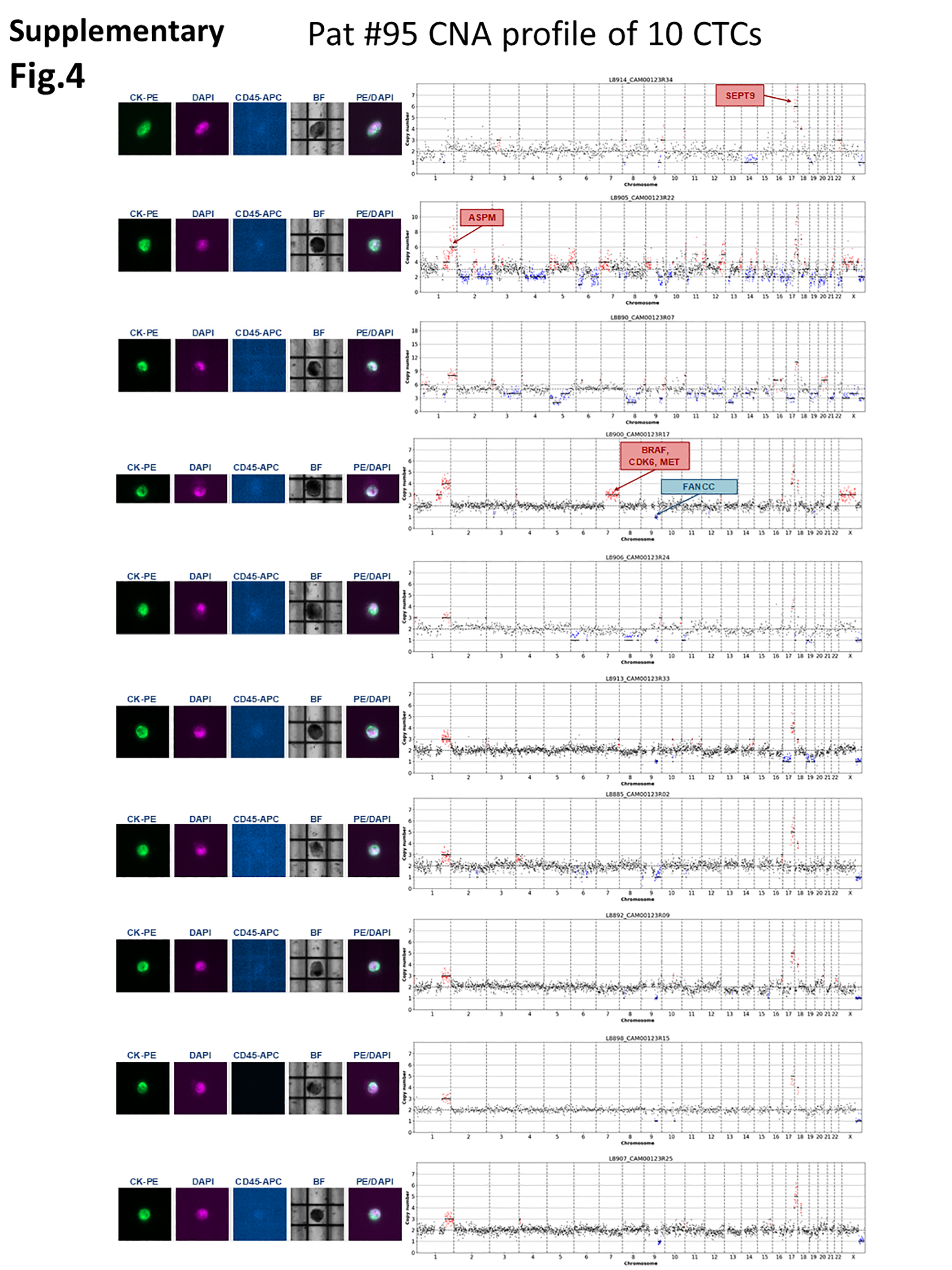

Supplement: Supplementary Figure 4 — Patient #95 single-CTCs images and CNA profiles. Cell gallery acquired by DEPArray, showing single fluorescent channels and overlays, and bright field (BF) images for 10 CTCs, paired with the corresponding CNA profile. [file Image_4.TIF]
